# An Account of a Child Who Drinks a Great Quantity of Water

**Published:** 1792

**Authors:** M. Vauquelin


XVI. *An Account of a Child who drinks a great Quantity of Water. By M.* Vauquelin.—Vide *La Medecine Eclairée par les Sciences physiques, ou Journal des Découvertes relatives aux differentes Parties de l'Art de Guerir; redigé par M.* Fourcroy. *Tome* III. 8vo. Paris, 1792.

WE have here another inſtance of polydipſia in addition to the two we have already recorded *. The ſubject of the preſent caſe is a boy, five years old, who is ſaid to be of a lively diſpoſition, and (this preternatural thirſt excepted) apparently in good health. His pulſe, we are told, beats from eighty to eighty-five times in a minute; and he reſpires from fifteen to eighteen times within the ſame period.

In the courſe of twenty-four hours, during which our author remained with him in a room, the temperature of which was from 50° to 55° of Fahrenheit's thermometer, he drank ten quarts of water, at about 50°, and voided twelve quarts of urine. He commonly ſleeps ten hours of every twenty four. In the day time he generally requires a ſupply of drink

* Vol. II. page 73.

every half hour; and at night his ſleep is interrupted once, at leaſt, in every hour by his thirſt, and an inclination to make water; and it is obſerved that, notwithſtanding theſe frequent interruptions of his ſleep, he every night voids urine in bed.

When he drinks it is with evident marks of greedineſs; his eyes and countenance are expreſſive of the comfort he experiences; and the moment he has done drinking he appears lively and happy. If drink be at any time refuſed him, when his inclination for it returns, he becomes affected with a tremulous motion of the heart, which ceaſes the moment he has drank; and ſo great is his eagerneſs to allay his thirſt, that he ſeizes with avidity any thing within his reach that has the appearance of liquor, and, if not prevented, will even drink his urine. Soon after he has drank he has a ſenſation of coldneſs, with a ſlight ſhivering; his countenance, at the ſame time, acquiring a bluiſh tint, and his breath feeling cool.

At the time this account was drawn up the patient had laboured under this complaint four months. It was firſt obſerved a little before the period of his being ſeized with the ſmall pox.

The urine he voided, while M. Vauquelin was with him, was as clear as water, but had a difagreeable fmell. It raifed the mercury in the thermometer to 100°. It did not fenfibly redden tincture of tournefol; and was rendered but in a flight degree turbid by the addition of lime water. Volatile alkali produced no change in it. In its fpecific gravity it did not fenfibly differ from water. On being expofed to the air it was obferved to be much fooner decompofed than human urine, in a healthy ftate, ufually is. This decompofition manifefted itfelf by a milky colour, and by a very difagreeable fmell. When expofed to heat, in an open veffel, it acquired a reddifh colour in proportion as it evaporated, and its difagreeable fmell was gradually diffipated. When about three fourths of it were evaporated it reddened tincture of tournefol; and, by completely evaporating it, our author obtained fixty-three grains of a refiduum which contained phofphorated foda, volatile alkali, a large proportion of fea falt, a mucous extract, and phofphoric acid in an uncombined ftate.

We have feen that this child drank ten quarts of water, the temperature of which was about 50°, in twenty-four hours, and that in the fame fpace of time he voided twelve quarts of urine at 100°. This great lofs of the principle of heat heat ſerves, in M. Vauquelin's opinion, to explain why the patient experiences coldneſs and ſhivering immediately after he has drank; and alſo why his breath is cold, and his face and lips appear of a violet colour.

As he voids by urine as much and even more than he drinks, and the diſcharge by the ſkin ſeems to be preternaturally diminiſhed, our author is diſpoſed to think that the exceſſive thirſt, in this caſe, may be occaſioned by ſome change that has taken place in the functions of the ſkin. This, he acknowledges, is merely an hypotheſis; but it is an hypotheſis, he obſerves, which would become realiſed, if, by reſtoring the perſpiration to its natural ſtate, the immoderate thirſt ſhould be relieved. He is aware, however, that ſeveral analogous facts muſt be collected, and compared with the preſent and the other few inſtances we have of this diſeaſe, before we can hope to aſcertain its cauſes. For this reaſon he earneſtly recommends it to his readers to avail themſelves of every opportunity that may occur to them of obſerving the phænomena of affections of this kind, and to mark with accuracy, in ſuch caſes, the temperature of the ſkin and urine, and likewiſe the ſtate of the pulſe and reſpiration.

